# Segmental uniparental disomy of chromosome 4 in a patient with methylmalonic acidemia

**DOI:** 10.1002/mgg3.1063

**Published:** 2019-12-02

**Authors:** Min Chen, Hu Hao, Hui Xiong, Yao Cai, Fei Ma, Congcong Shi, Xin Xiao, Sitao Li

**Affiliations:** ^1^ Department of Pediatrics The Sixth Affiliated Hospital of Sun Yat‐Sen University Guangzhou China; ^2^ Inborn Errors of Metabolism Laboratory The Sixth Affiliated Hospital of Sun Yat‐Sen University Guangzhou China

**Keywords:** metabolic crisis, methylmalonic acidemia, *MMAA* gene, segmental uniparental disomy, SNP array

## Abstract

**Background:**

Methylmalonic acidemia (MMA) is an autosomal recessive genetic disorder involving the metabolism of organic acids.

**Methods:**

Here, we report the case of a patient who developed acute metabolic crisis after vaccination and was diagnosed with *cblA* type MMA after hospitalization.

**Results:**

Further examination revealed a homozygous pathogenic variant in the *MMAA* gene that caused the disease in the patient but did not conform to Mendelian inheritance. Using chromosomal microarray analysis, maternal uniparental disomy (UPD) was found on chromosome 4q26‐q35.2 of the patient. The *MMAA* gene of the patient was inherited only from the mother and carried the same pathogenic variant on both alleles of chromosome 4. *MMAA* gene expression levels in whole blood were detected by real‐time PCR.

**Conclusion:**

The nonsense pathogenic variant, NM_172250.2:c.742C>T (p.Gln248*), carried by the patient leads to a premature termination of transcription of the gene, thereby resulting in partial loss of protein function while retaining some others. Segmental UPD 4 is rare, and to our knowledge, has not been reported previously.

## Graphical abstract

We report a case that developed acute metabolic crisis after vaccination, and was diagnosed with cblA‐type of methylmalonic acidemia (MMA) after hospitalization. Using chromosomal microarray analysis, maternal uniparental disomy (UPD) was found in chromosome 4q26‐q35.2 of the patient. MMA due to UPD 4 had not been reported earlier.

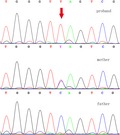

## 1 INTRODUCTION

Methylmalonic acidemia (MMA), an autosomal recessive genetic disease, which affects organic acid metabolism, is mainly caused by a defective methylmalonyl‐CoA mutase or abnormal metabolism of coenzyme cobalamin (Vitamin B_12_) (Deodato, Boenzi, Santorelli, & Dionisi‐Vici, 2006; Oberholzer, Levin, Burgess, & Young, 1967). Based on varying blood homocysteine levels in patients with MMA, the disease may be categorized as either MMA or MMA combined with homocysteinemia (Yang et al., 2006). In China, MMA combined with homocysteine accounted for 60%–80% of the total number of patients with MMA. These included four subtypes, *cblC* type (MIM 277400), classic *cblD* type (MIM 277410), *cblF* type (MIM 277380), and *cblJ* type (MIM 614857) (Lin et al., 2018). Among them, *cblC* subtype is the most common (Wang et al., 2010). Isolated MMA, without homocysteine, includes four subtypes, mut (0)/mut‐type (MIM 251000), *cblA* type (MIM 251100), *cblB* type (MIM 251110), and *cblD* variant 2 type (MIM 277410) (Lin et al., 2018). Clinical symptoms of MMA typically manifest within the first year of life, although some children have late‐onset clinical symptoms when they are several years old or even into their teens (Haarmann et al., 2013; Lerner‐Ellis et al., 2004). The clinical manifestations of MMA include multi‐system injuries of the skin, blood, and nerves, among which brain nerve damage is more severe and the rate of disability is high (Zhou, Cui, & Han, 2018). Although the clinical manifestations in patients with MMA that have different etiologies are similar, their prognoses maybe different due to individual differences, with high mortality and disability rates in severe cases (Liu et al., 2018).

Here, we report on a patient diagnosed with MMA. In order to identify the etiology, we employed exome sequencing and it was identified that the patient had a pathogenic homozygous pathogenic variant, NM_172250.2:c.742C>T (p.Gln248*), in the *MMAA* gene. And chromosomal microarray analysis by single nucleotide polymorphism (SNP) array technology showed the associated pathogenic variant was non‐Mendelian, and segmental uniparental disomy (UPD) of chromosome 4 was seen in the progeny. Segmental UPD 4 is rare, and MMA due to segmental UPD 4 has not been reported previously.

## 2 MATERIALS AND METHODS

### 2.1 Clinical report

The patient was an 8‐month‐old girl with no report of abnormalities or delays in early motor milestones prior to vaccination. Approximately an hour after vaccination with the hepatitis B and meningococcal group A vaccines, the patient presented with vomiting, fever, cough, and opisthotonos. The patient's condition did not improve after upper respiratory tract treatment at a local hospital, and subsequently worsened after 10 days. She was lethargic and refused to eat, therefore, she was transferred to a superior hospital for treatment and was diagnosed with viral encephalitis and possible genetic metabolic disease. After treatment with oseltamivir, methylprednisolone, and immunoglobulin, fever reduced and her responses improved. Gas chromatography‐mass spectrometry (GS‐MS) and tandem mass spectrometry (MS/MS) performed on urine samples suggested the possibility of MMA. The patient was then transferred to our hospital for further treatment.

Routine laboratory examinations included blood and urine biochemistry. No obvious abnormality was noted in liver function. Plasma homocysteine was in the normal range: 4.46 µmol/L (normal range 0–15 µmol/L). Urine GC‐MS confirmed elevated methylmalonic and methylcitrate, confirming the diagnosis of isolated MMA. The concentrations of blood propionyl carnitine (C3) and the C3 to acetylcarnitine (C2) ratio (C3/C2) were analyzed using MS/MS. C3 was 6.853 µmol/L (normal range 0.3–3 µmol/L) and C3/C2 was 0.85 µmol/L (normal range 0.02–0.16 µmol/L), suggesting propionic or MMA. A head MRI showed suspicious spotty and linear T2‐FLAIR hyperfocal lesions in the white matter of the brain. Both lateral ventricles and the third ventricle were dilated. The cerebral sulcus was widened.

Then she was treated with an intramuscular injection of vitamin B_12_ (1 mg/day), an intravenous infusion of l‐carnitine (100 mg kg day^−1^), a low protein diet (1–2 g kg day^−1^), and a special formula free from methionine, valine, and threonine. After treatment, she improved, and long‐term oral administration of l‐carnitine (1 g/day) and Cyanocobalamin (0.5 mg/day), along with maintenance of the low protein diet and formula continued. At the 6‐month follow‐up, the patient was in stable condition with no obvious abnormalities in growth and development. And C3, C3/C2 level decreased and only a small amount of methylmalonic acid was excreted in urine organic acid.

### 2.2 Molecular genetic testing methods

This study was performed in accordance with the principles of the Declaration of Helsinki and was approved by the Ethics Committee of the Sixth Affiliated Hospital, Sun Yat‐sen University (Approval number: 2017ZSLYEC‐105).Written informed consent was obtained from the parents of the patient. After obtaining written informed consent, peripheral blood was drawn from the patient and her parents using the Agilent capture kit and Illumina SBS kit. High‐throughput sequencing was performed on the Illumina HiSeq platform and the Clinical Sequence Analyzer (CSA) from WuXi NextCODE was used for quantitative analysis. The Sentieon software suite was mainly used for secondary analysis of the sequencing data. Sequencing data were compared to the UCSC hg19 reference genome by Sentieon BWA. Variants were annotated with a process developed using WuXi NextCODE, and each variant was evaluated using the CSA, developed and validated by WuXi NextCODE. Sequencing results were compared with DECIPHER (https://decipher.sanger.ac.uk), DGV (http://dgv.tcag.ca/dgv/app/home), ClinVar (http://www.ncbi.nlm.nih.gov/clinvar/), and a local database. After obtaining the *MMAA* gene pathogenic variant, Sanger sequencing was performed to verify the pathogenic locus of the single gene. GenBank reference sequence and version number:*MMAA* (GeneID: 166785; NM_172250.3).

### 2.3 The mRNA level testing methods

*MMAA* gene expression levels in whole blood samples from the patient, mother of the patient, and normal individuals were detected by real‐time PCR. Primers for the pre‐ variant and mutated sequences were designed to cover the variant site, c.742C>T. Fluorescence quantitative PCR (SYBR Green method) was used to detect the mRNA expression level of *MMAA* gene. The pre‐sequence and post‐sequence of the variant site c.742C>T were amplified respectively, and the primer sequences respectively were: (a) Sequence name: c.742C>T, forward primer: 5’‐AGGAGTTAGCCCAGGTGCTTC‐3’, reverse primer: 5’‐CCAGACAATCCTACTCGAAATGCT‐3’; (b) Sequence Name: c.742C>T, forward primer: 5’‐AGGAGATGAGCTGCAGGGTATC‐3’, reverse primer: 5’‐TATCCTTCGAGCTGGCACAA‐3’. Forty replicates were used and all amplified fragments were 103 bp. The relative expression of the sample was calculated using the $2^{-\Delta \Delta C_{T}}$ method, where Δ*C_T_* equals the target gene *C_T_*‐internal reference gene *C_T_*, and ΔΔ*C_T_* equals Δ*C_T_* of patient group − Δ*C_T_* of the control group/maximum value. *C_T_* is the cycle number that correlates to the fluorescence signal as it reaches the set domain value.

## 3 RESULTS

**Figure 1:**
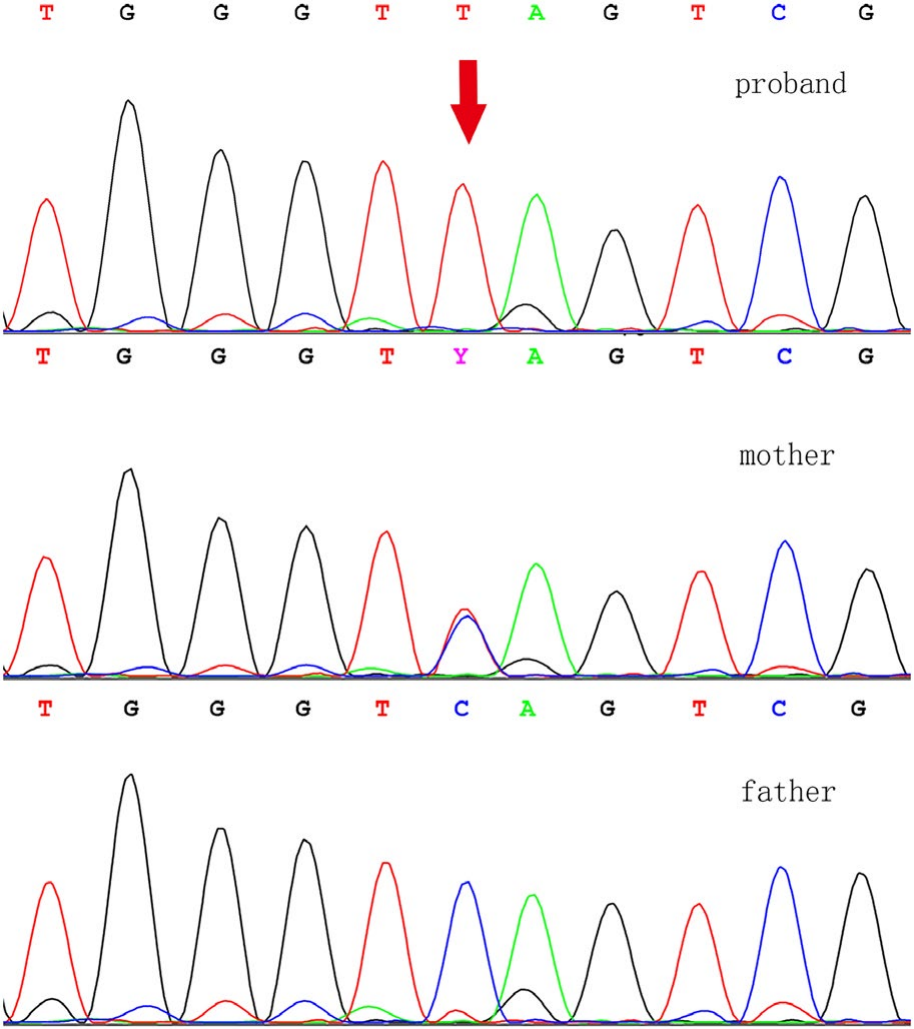
Proband:*MMAA* gene sequence in the patient c.742 C>T homozygous variant; mother:*MMAA* gene sequence in the mother c.742 C>T heterozygous variant; father: *MMAA* gene sequence in the father no pathogenic variant

**Figure 2:**
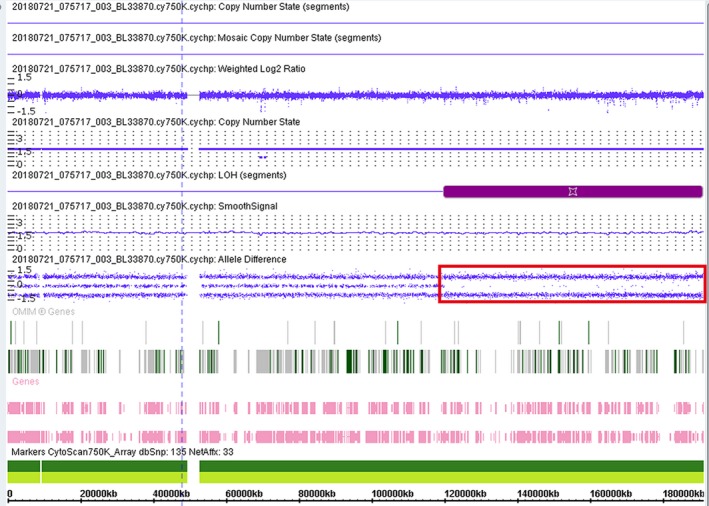
The red box shows the region of chromosome 4q26q35. 2 that had a loss of heterozygosity

The NM_172250.2:c.742C>T (p.Gln248*) pathogenic variant in the *MMAA* gene was found in the patient and mother, confirming *cblA* type MMA. In the sequencing data of the family, the patient was diagnosed with a homozygous pathogenic variant, the mother with a heterozygous pathogenic variant, and the father had no pathogenic variants (Figure 1). The Affymetrix CytoScan 750K SNP‐array (ThermoFisher) was used for chromosomal microarray analysis, and the results revealed segmental UPD in chromosome 4q26‐q35.2 of the patient. This is the first case of *cblA* type MMA caused by a homozygous pathogenic variant in the *MMAA* gene from segmental UPD 4. And this segmental UPD is an isodisomy (inheritance of two copies of a single chromosome homolog from one parent). The molecular karyotype was 46,XX,arr[hg19] 4q26q35.2 (119,806,937–190,921,709)x2 hmz (Figure 2). SNP array analysis of the patient revealed that the q26q35.2 area of chromosome 4 has a 71.1 Mb loss of heterozygosity, and contains 50 genes associated with an autosomal recessive genetic disease (AR), for example, AGA (613228), TENM3 (610083), TRAPPC11 (614138), etc. Determining whether these lead to disease would require further follow‐up, and the effect of the loss of heterozygosity also remains unclear.

**Figure 3:**
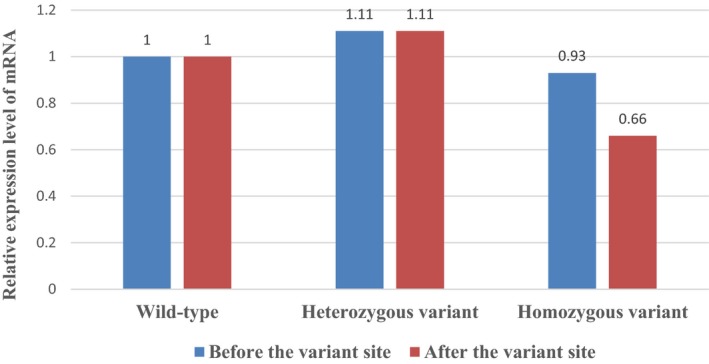
Expression levels of the *MMAA* gene in blood samples compared between wild type, heterozygous c.742 C>T variant, and homozygous c.742 C>T variant

Expression levels of the *MMAA* gene in whole blood samples were detected by real‐time PCR. The results showed that the relative expression of the pre‐variant sequence and sequences after the pathogenic variant of the *MMAA* gene was 1.000 in the homozygous sample. The relative expression was 1.1865 and 1.1674 in the pre‐variant and post variant sequence, respectively, in the maternal mutant. The relative expression was 1.0669 and 1.5052 in the pre‐variant and post variant sequence, respectively, in wild‐type samples (Figure 3).

## 4 DISCUSSION

MMA is a common organic acidemia with regional differences in its incidence, ranging from 1:48,000 to 1:250,000 and its incidence in China remains unclear (Liu et al., 2012; Yang et al., 2008). In China, most of the reported cases of isolated MMA are mut (0)/mut‐type, whereas *cblA* type is the least common. Only two patients with MMA have been previously diagnosed with *cblA* type (Liu et al., 2015; Sun et al., 2015).

The *MMAA* geneis approximately 17.1‐kb long, located on chromosome 4q31.1‐q31.2, contains seven exons, and encodes a protein that is 418 amino acids (Keyfi et al., 2016). Over 60 pathogenic variants in the *MMAA* gene have been reported that lead to isolated MMA. Some of these pathogenic variants are known to be unique to a particular race (Dempsey‐Nunez et al., 2012; Keyfi et al., 2016; Plessl et al., 2017). For instance, c.433C>T is common in Europe, and is always accompanied by SNP c.820‐110A>G (Lerner‐Ellis et al., 2004), while c.503del is common among patients in Japan (Yang et al., 2004).

Dorchy (1995) has reported a case of Neonatal diabetes mellitus caused by congenital absence of beta cells associated with MMA due to UPD of chromosome 6. In the present case, high‐throughput sequencing results showed that the patient had *cblA* type MMA and the NM_172250.2:c.742C>T pathogenic variant was a homozygous pathogenic variant, inherited from the mother. Further chromosomal microarray analysis revealed that all sites of chromosome 4q26‐q35.2 were homozygous, although the copy number was normal diploid. Analysis confirmed the genetic relationship of the patient to her parents, confirming that chromosome 4q26‐q35.2 of the patient was maternal UPD. UPD can occur during gametic meiosis or mitosis of the fertilized egg, and is more common in the imprinting region of chromosome 15q11.2‐q13.1, such as inPrader‐Willi/Angelman syndrome (Morandi et al., 2015).

Here, we have found the first case of *cblA*‐type MMA caused by the NM_172250.2:c.742C>T (p.Gln248*) homozygous pathogenic variant of the *MMAA* gene on segmental UPD 4 with the molecular karyotype of 46,XX,arr[hg19]4q26q35.2 (119,806,937–190,921,709)x2 hmz. The nonsense pathogenic variant, NM_172250.2:c.742C>T (p.Gln248*), carried by the patient is associated with premature termination of the gene, synergistically the decrease trend in gene expression, could lead to partial lost function and decreased expression of the protein. When the human body is in stress, caused by fever, improper diet, fatigue, drugs, and hunger, its metabolic homeostasis may be destroyed causing acute onset of the disease. In general, the clinical phenotype manifested in patients is relatively mild, and prognosis is mostly good, as shown in previous studies (Devi & Naushad, 2017; Vatanavicharn et al., 2012).

UPD is a chromosomal variation in which two homologous chromosomes or chromosomal regions are inherited only from the parent, and can be classified into isodisomy (inheritance of two copies of a single chromosome homolog from one parent) and heterodisomy (inheritance of a pair of homologous chromosomes from one parent). In many UPD cases, chromosomes of both isodisomy and heterodisomy may be present (Eggermann, Soellner, Buiting, & Kotzot, 2014). Trisomy rescue, gamete complementation, monosomy rescue, and post‐fertilization mitotic error are the candidate mechanisms underlying UPD formation. Our current case was of maternal UPD. Since the imprinted gene is expressed in a way specific to parental origin, maternal UPD leads to over expression of maternal genes; there was no paternal gene expression on the affected chromosomal regions. Meiotic errors that occurred in the process of oogenesis, especially nondisjunction of two homologous chromosomes and the premature separation of sister chromatids, produce disomic oocytes. It is worth noting that disomic oocytes can lead to further trisomy rescue and gamete complementation, and the two can cause UPD (Matsubara, Kagami, & Fukami, 2018).

The first reported case of isodisomy unmasking a mutant recessive allele was described by Spence and co‐workers in 1988 (Spence et al., 1988). They described a small child with CF (cystic fibrosis) who had received two copies of the same chromosome 7 with a CFTR (cystic fibrosis transmembrane conductance regulator) variant from her carrier mother, and no contribution from her non‐carrier father. Since this initial report, more than 50 cases of recessive disorders have been found to be associated with UPD (Engel, 2006; Kotzot, 2004). UPD as one kind of cause for autosomal recessive diseases and be more common than previously thought. When looking from a genome‐wide perspective (Sasaki, Mishima, Miura, & Yoshiura, 2013), an estimated rate of segmental UPD is thought to be at 0.578% based on a UPD screening, or 0.026% based on autosomal chromosome pairs investigated in the general population. These data imply the possibility of hidden segmental UPD in the general population (Sasaki et al., 2013).

Unless there is clinical evidence of other cytogenetic abnormalities, UPD can be difficult to detect by simple karyotype or copy number analysis. Identification of UPD by standard sequencing also proved inadequate. For the detection of UPD, chromosomal microarray analysis, such as SNP array genotyping or microsatellite analysis, of DNA samples from patients and their parents are currently the preferred detection methods. In addition, SNP analysis plus comparative genomic hybridization array is a very effective method to screen uniparental isodisomy throughout the entire genome (Dawson et al., 2011; Joshi et al., 2016).

UPD is a prime topic in the field of human genetics and reproductive medicine. A previous study had shown that the incidence of UPD in live births was 1 in 3,500. The frequency of such meiotic error increases with age (Herbert, Kalleas, Cooney, Lamb, & Lister, 2015), therefore, the incidence of UPD in older mothers is likely to be higher (Eggermann et al., 2015; Matsubara, Murakami, Nagai, & Ogata, 2011). UPD frequency is then likely to increase in the near future, as the average age of fertility is increasing in many countries (Eggermann et al., 2015; Matsubara et al., 2011).

This report suggests that UPD may be a cause of autosomal recessive disorders, especially when patients have a rare homozygous pathogenic variant. Here, we have reported the first case of *cblA* type MMA caused by segmental UPD 4. The segmental UPD 4 result in a NM_172250.2:c.742C>T (p.Gln248*) homozygous pathogenic variant of the *MMAA* gene. The chromosome molecular karyotype is 46,XX,arr[hg19]4q26q35.2 (119,806,937–190,921,709)x2 hmz. The effects of this variant need to be further studied in future. We suggest that SNP or microsatellite genotyping would be useful in diagnose patients with UPD.

## CONFLICTS OF INTEREST

All authors have no conflicting financial interests to declare.

## AUTHOR CONTRIBUTIONS

Fei Ma and Congcong Shi performed analysis of the sequence data. Min http://www.wanfangdata.com.cn/details/detail.do?_type=perio%26xml:id=syeklczz201621010 provided molecular analysis other than genetic sequence analysis and prepared the manuscript. Yao Cai provided the clinical follow‐up. Hu http://www.wanfangdata.com.cn/details/detail.do?_type=perio%26xml:id=syeklczz201621010 and Hui Xiong edited the paper.
